# Light-induced formation of partially reduced oxygen species limits the lifetime of photosystem 1-based biocathodes

**DOI:** 10.1038/s41467-018-04433-z

**Published:** 2018-05-17

**Authors:** Fangyuan Zhao, Steffen Hardt, Volker Hartmann, Huijie Zhang, Marc M. Nowaczyk, Matthias Rögner, Nicolas Plumeré, Wolfgang Schuhmann, Felipe Conzuelo

**Affiliations:** 1 Analytical Chemistry – Center for Electrochemical Sciences (CES), Ruhr-Universität Bochum, Universitätsstr. 150, Bochum, 44780 Germany; 2 Center for Electrochemical Sciences – Molecular Nanostructures, Ruhr-Universität Bochum, Universitätsstr. 150, Bochum, 44780 Germany; 3 Plant Biochemistry, Ruhr-Universität Bochum, Universitätsstr. 150, 44780 Bochum, Germany

## Abstract

Interfacing photosynthetic proteins specifically photosystem 1 (PS1) with electrodes enables light-induced charge separation processes for powering semiartificial photobiodevices with, however, limited long-term stability. Here, we present the in-depth evaluation of a PS1/Os-complex-modified redox polymer-based biocathode by means of scanning photoelectrochemical microscopy. Focalized local illumination of the bioelectrode and concomitant collection of H_2_O_2_ at the closely positioned microelectrode provide evidence for the formation of partially reduced oxygen species under light conditions. Long-term evaluation of the photocathode at different O_2_ concentrations as well as after incorporating catalase and superoxide dismutase reveals the particularly challenging issue of avoiding the generation of reactive species. Moreover, the evaluation of films prepared with inactivated PS1 and free chlorophyll points out additional possible pathways for the generation of oxygen radicals. To avoid degradation of PS1 during illumination and hence to enhance the long-term stability, the operation of biophotocathodes under anaerobic conditions is indispensable.

## Abstract (web summary)

Photobiodevices use photosynthetic proteins such as those of the photosystem 1 (PS1) to enable light-induced charge separation, but they suffer from limited long-term stability. Here authors employ scanning photoelectrochemical microscopy on a PS1 biocathode and find that several pathways generate oxygen radicals.

## Introduction

The integration of isolated photosynthetic protein complexes such as photosystem 1 (PS1) or photosystem 2 (PS2) into semiartificial photoelectrochemical devices requires immobilization strategies that allow optimized electron transfer while ensuring its adequate stability. The design-engineered electron-transfer chains for an efficient coupling of photosynthetic proteins to electrode surfaces were demonstrated for PS1^1–10^, PS2^11–15^, and bacterial reaction centers^16, 17^. However, light-induced damage of photosynthetic protein complexes in semiartificial assemblies causes fast degradation together with a drop in activity. Consequently, the potential applicability of biohybrid devices is considerably limited^18, 19^.

Environmental as well as light-induced stress in plants creates an imbalance between the produced reactive oxygen species (ROS) and scavenging antioxidant systems^20^. PS2 has been considered the primary target for photoinhibition^21^ and much is known about the mechanism of damage and repair in vivo^21–26^. In contrast, PS1 presents a considerably longer life time in vivo^27^ and has been considered to be less sensitive to light stress than PS2^22, 28^. However, PS1 is similarly susceptible to environmental stress^29, 30^, with ROS causing inactivation of the protein complex^22, 23, 31, 32^. Moreover, since damaged PS1 is not repaired as PS2^33^, PS1 damage seems to be irreversible and may have a much stronger impact on the survival of plants than PS2 photoinhibition^23^.

In photobioelectrochemical devices that utilize O_2_ as terminal electron acceptor, the generation of ROS is inherently associated. Since the reduction of O_2_ to H_2_O requires four electrons, the stepwise production of partially reduced intermediates prevails. The initial step in the formation of partially reduced oxygen species is the generation of the superoxide radical (O_2_^•−^) by a one electron reduction of molecular oxygen. The cascade of reactions continues with dismutation of O_2_^•−^ leading to the production of H_2_O_2_ and the formation of the hydroxyl radical (HO^•^) by a one electron reduction process^20, 25, 34^. Moreover, a kinetically hindered uptake of electrons from the F_B_^−^ site of PS1 can result in back-reactions leading to the formation of chlorophyll in an excited triplet state, able to react with O_2_ under formation of singlet oxygen, O_2_(^1^Δ_g_)^35^. All these highly reactive species can easily disrupt any biological assembly^36, 37^.

Many PS1-based semiartificial devices make use of redox mediators to enhance the electron-transfer rate from the photosynthetic protein complex to O_2_. Viologen derivatives exhibit sufficiently negative redox potentials and are widely used as redox mediators for the efficient electrocatalytic reduction of O_2_^38, 39^. However, the increased electron-transfer rates are also associated with an increased generation of partially reduced oxygen species. Particularly, methyl viologen (MV^2+^) is known for its herbicidal activity in plants due to the production of partially reduced ROS, with H_2_O_2_ as the major product in aqueous solutions, and peroxidation of lipid constituents eventually leading to necrosis^39^. MV^2+^ effectively scavenges the high-energy electrons from PS1. A voltammetric study about MV^2+^-mediated reduction of O_2_ suggested^39, 40^ that the generated methyl viologen radical cation (MV^+•^) reacts efficiently with O_2_ to generate the superoxide radical (Eq. (1)):

1 $${\mathrm{MV}}^{ + \bullet } + {\mathrm{O}}_2 \to {\mathrm{MV}}^{2 + } + {\mathrm{O}}_2^{ \bullet - }\quad {{k}}_{\mathrm{f}} = 6 \times 10^9{\kern 1pt} {\mathrm{M}}^{ - 1}{\kern 1pt} {\mathrm{s}}^{ - 1}$$

Subsequently, the generated ${\mathrm{O}}_2^{ \bullet - }$ may dismutate or further react with MV^+•^ with the eventual generation of H_2_O_2_ (Eqs. (2) and (3)):

2 $$2{\mathrm{O}}_2^{ \bullet - } + {\mathrm{2H}}^ + \to {\mathrm{O}}_2 + {\mathrm{H}}_{\mathrm{2}}{\mathrm{O}}_{\mathrm{2}}\quad {{k}}_{\mathrm{f}} = 1.3 \times 10^6{\kern 1pt} {\mathrm{M}}^{ - 1}{\kern 1pt} {\mathrm{s}}^{ - 1}$$

3 $${\mathrm{MV}}^{ + \bullet } + {\mathrm{O}}_2^{ \bullet - } + {\mathrm{2H}}^ + \to {\mathrm{MV}}^{2 + } + {\mathrm{H}}_2{\mathrm{O}}_2\quad {k}_{\mathrm{f}} = 6.5 \times 10^8{\kern 1pt} {\mathrm{M}}^{ - 1}{\kern 1pt} {\mathrm{s}}^{ - 1}$$

Therefore, the occurrence of H_2_O_2_ can be considered as evidence for the generation of partially reduced oxygen species causing deleterious processes of PS1-based biophotocathodes under aerobic conditions. Our aim was to implement an analytical system for the simultaneous evaluation of O_2_ consumption and associated H_2_O_2_ production under irradiation and to gain deeper understanding on light-induced stress.

As the integration of isolated photosynthetic protein complexes with electrodes decreases their stability and prevents biological repair mechanisms, the effect of reactive species for semiartificial devices is even more delicate. While competing charge transfer pathways and short circuits at the integrated chlorophyll molecules constituting antenna complexes further restrain the efficiency of PS2-based biodevices^41, 42^, the immobilization of isolated PS1 at electrodes has shown as well a rather limited stability upon light irradiation^18, 19, 29^. However, the long-term stability of PS1-based photoelectrodes has been commonly underestimated and much less is known about inactivation processes and ROS generation at devices using isolated PS1 coupled to electrode surfaces. Particularly, under aerobic conditions and when MV^2+^ is used as an electron acceptor, the fast electron transfer to O_2_ is unequivocally causing the generation of partially reduced oxygen species^39^, leading to an eventual loss in PS1 activity. The deleterious effect of partially reduced oxygen species generated under light is therefore also of particular importance for PS1-based photobioelectrodes operated under aerobic conditions, an effect which until now has been suggested but not extensively evaluated because of the lack of experimental evidence.

Scanning electrochemical microscopy (SECM) is a scanning probe technique for the visualization of local electrochemical activity. An accurately positioned microelectrode tip is used as a probe for the evaluation of electrochemical processes or collection of species of interest evolved at the sample. Particularly for the analysis of photoactive materials, scanning photoelectrochemical microscopy (SPECM) was suggested combining SECM with a light source for the controlled and focalized illumination of the sample^43, 44^.

We present an in-depth evaluation of a highly efficient photobiocathode^18^ comprising isolated PS1 embedded within an Os-complex-modified polymer. SPECM is used for investigation of light-induced stress at the PS1-based photocathode and the concomitant generation of partially reduced oxygen species supposedly involved in PS1 degradation. Evidence for the formation of partially reduced oxygen species under illumination of PS1/redox polymer-modified photobiocathodes is demonstrated by in situ collection of H_2_O_2_ at the SPECM tip. Simultaneous localized irradiation of the PS1-based photocathode and collection of H_2_O_2_ at different O_2_ concentrations and in the presence of scavenging enzymes, i.e., catalase (Cat) and superoxide dismutase (SOD), is presented. Moreover, by studying inactivated PS1 and free chlorophyll immobilized within the Os-complex-modified polymer, previous observations at PS2-based bioanodes concerning the light-induced generation of partially reduced oxygen intermediates at chlorophyll pigments were confirmed^41, 42^.

## Results

### Electron transfer in PS1/Os-P with O_2_ as terminal acceptor

**Fig. 1:**
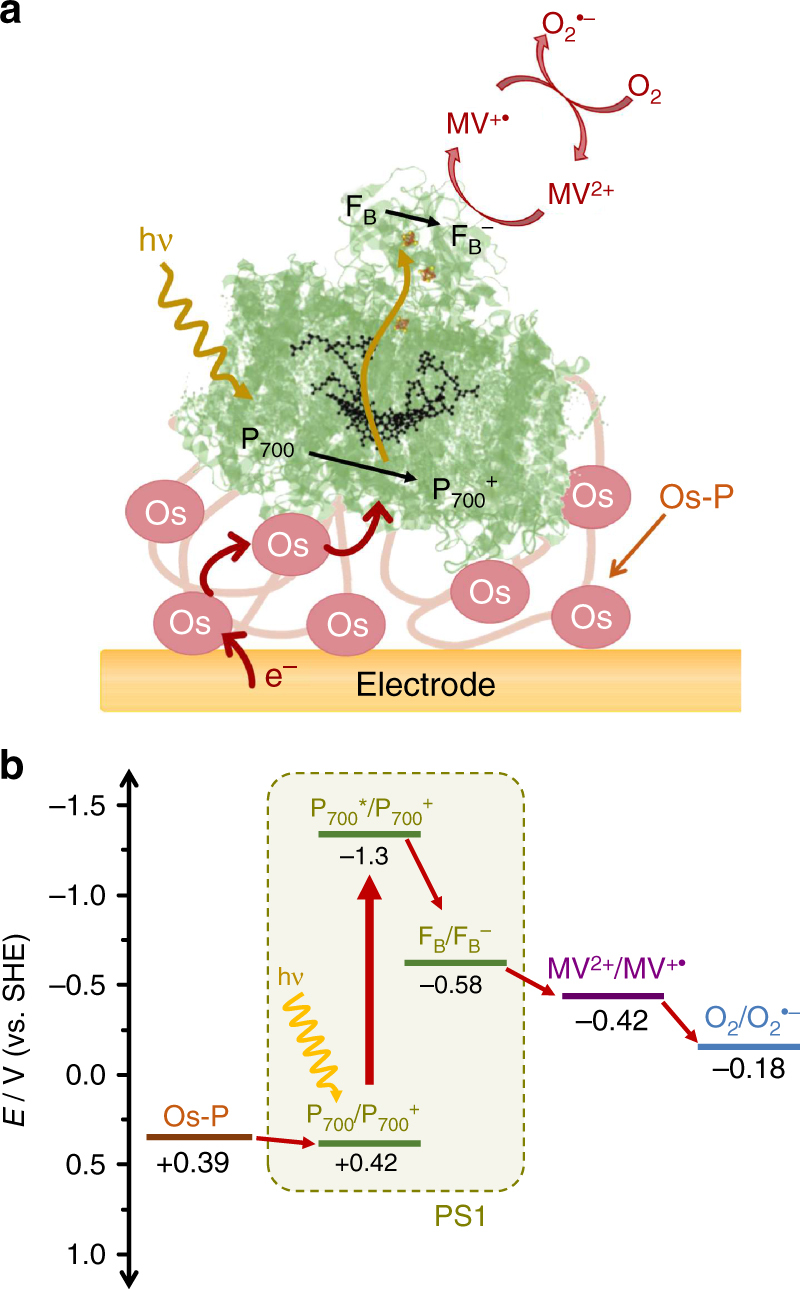
Overview of the charge transfer pathway in the PS1/Os-complex-modified redox polymer-based photocathode. **a** Schematic representation of the PS1 photocathode. The redox polymer acts simultaneously as immobilization matrix and as a mediator for the reduction of the photo-oxidized P_700_^+^ site at PS1. MV^2+^ is used as electron scavenger in solution for the uptake of electrons from the reduced Fe-S cluster F_B_^−^ at the stromal side of PS1. The efficient reduction of O_2_ in solution by the generated MV^+•^ allows fast regeneration of MV^2+^ in solution with the concomitant generation of superoxide radicals. (P_700_*: photo-excited P_700_ special chlorophyll pair). **b** Energy levels for the terminal cofactors involved in the electron-transfer sequence of PS1 and formal potentials of the Os-complex-modified polymer (Os-P), methyl viologen, and the O_2_/O_2_^•−^ couple at pH 7.0 (25 °C)

By integration of isolated PS1 within an optimized redox hydrogel and working under experimental conditions that ensure that the process is limited only by the availability of O_2_ as terminal electron acceptor, benchmark electron-transfer rates were achieved that are even capable of outperforming the rates observed in natural photosynthesis^18^. For the evaluation of the formation of partially reduced oxygen species and protein stability in semiartificial devices, we selected this very same PS1-based photocathode that has been previously optimized in terms of electronic communication with the electrode surface (Fig. 1a). The selected biodevice is a highly efficient assembly of PS1 integrated within an Os-complex-modified redox polymer acting as an immobilization matrix and as redox mediator for the transfer of electrons from the electrode surface to the PS1 moieties. As illustrated in the corresponding energy level diagram in Fig. 1b, PS1 performs charge separation upon light absorption, and after internal electron transfer, a final long-lived state with a charge recombination lifetime of about 20 ms is obtained^3^. In this state, the two opposite redox sites at PS1 are the photooxidized special chlorophyll pair P_700_^+^ at the luminal side and the reduced terminal Fe-S cluster F_B_^−^ at the stromal side. The Os-complexes in the redox hydrogel donate electrons for the reduction of P_700_^+^, while the high-energy electron is scavenged by the redox mediator MV^2+^ followed by the reduction of O_2_ as terminal electron acceptor. The fast process of O_2_ reduction by the generated MV^+•^ allows, on the one hand, a fast regeneration of the redox mediator in solution for the efficient uptake of electrons from the F_B_^−^ site, thus ensuring high photocurrents. However, on the other hand, superoxide radicals are simultaneously generated, which can also react with MV^+•^ or dismutate, leading to the eventual generation of H_2_O_2_. This electrochemical pathway leads to the generation of partially reduced ROS in the surroundings of the immobilized protein complexes and may have a deleterious effect leading to the possible inactivation of PS1 under illumination.

### SPECM evaluation of produced H_2_O_2_ at the PS1 photocathode

**Fig. 2:**
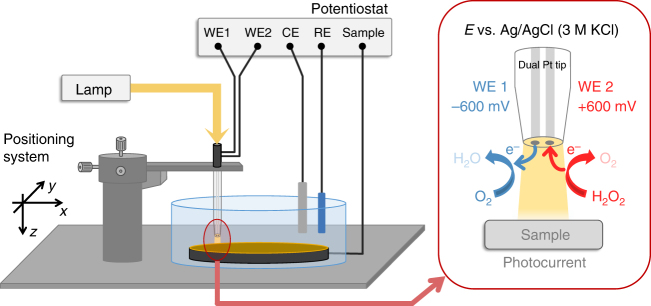
The PS1/redox polymer-based photocathode is investigated using SPECM. Representation of the SPECM setup. CE: counter electrode, RE: reference electrode, WE: working electrode. Right: dual Pt microdisk tip for the simultaneous collection of O_2_ and H_2_O_2_ while allowing localized irradiation of the sample through the glass sheath of the positioned dual microelectrode tip

A dual Pt microdisk electrode was used as electrochemical probe in an SPECM setup, allowing multiple parameters to be simultaneously monitored in a localized space. As depicted in Fig. 2, the SPECM setup allowed to monitor the photocurrent from the sample electrode, using the glass sheath of the dual microelectrode tip as extended light guide for the localized and controlled irradiation of the PS1 photocathode. Simultaneously, O_2_ consumption was monitored by detecting the O_2_ reduction current at one of the Pt microdisk electrodes, while in parallel the oxidation of H_2_O_2_ was measured at the second Pt microdisk electrode allowing to collect H_2_O_2_ generated at the sample. The dual microdisk electrodes were fabricated to minimize any potential crosstalk interference between the two disk electrodes (see Supplementary Fig. 1 and Methods section). The fast electrocatalytic conversion of the products at the selected applied potentials ensures diffusion-limited processes. Moreover, the tip-to-sample distance (typically 10 µm) was kept below one tip radius (Pt microdisk, *a* ≈ 12.5 µm) for the fast and efficient collection of evolved H_2_O_2_.

### Partially reduced oxygen species generated under light

**Fig. 3:**
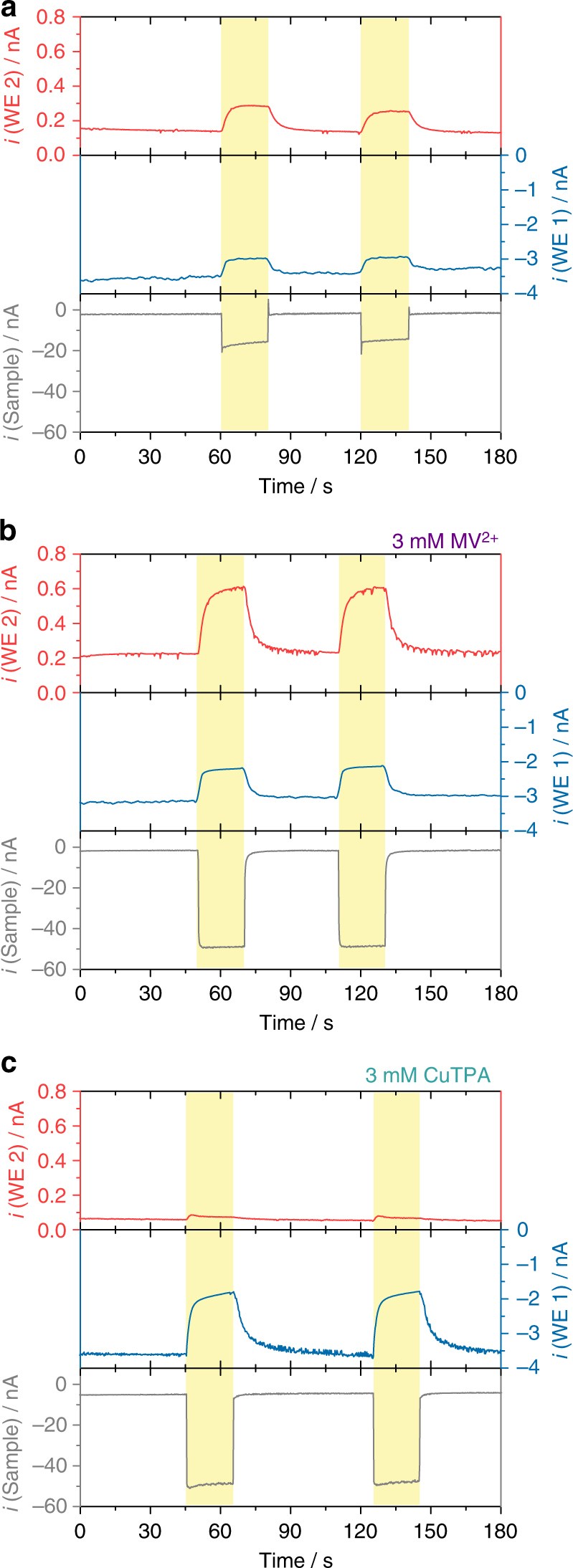
Evidence for the generation of partially reduced oxygen species under irradiation. Simultaneous photochronoamperometric responses recorded during evaluation of a PS1/Os-complex-modified redox polymer photocathode on a gold electrode surface (sample) using a dual Pt microelectrode tip: **a** air-equilibrated buffer, **b** in the presence of 3 mM MV^2+^, **c** in the presence of 3 mM CuTPA. The sample was polarized at 0 mV while the Pt disk microelectrodes were polarized at −600 mV (WE 1) and +600 mV (WE 2) vs. Ag/AgCl/3 M KCl. Electrolyte: 0.2 M citrate-phosphate buffer, pH 7.0. Tip-to-sample distance: 10 μm. The sample was locally irradiated (white light, 280 mW cm^−2^) for periods of 20 s through the glass sheath of the positioned dual Pt microdisk electrode, as indicated by the yellow shaded regions in the graphs

The PS1-based photocathode was investigated using SPECM by performing photochronoamperometric measurements in ambient air-equilibrated solutions and working at an incident illumination intensity that ensures light saturation of the immobilized PS1^18, 45^. First, the PS1/Os-complex-modified redox polymer was analyzed in the absence of any redox mediator in solution using dissolved O_2_ as electron acceptor. The obtained results for the simultaneous measurement of sample photocurrent together with the O_2_ and H_2_O_2_ collection at the dual Pt microdisk electrode are summarized in Fig. 3a. The photocurrent response obtained at the sample (Fig. 3a, gray trace) exhibited an increased cathodic current upon irradiation due to the reduction of the photo-oxidized P_700_^+^ site of PS1 by the Os-complexes in the redox hydrogel. Owing to the highly negative formal potential of the terminal Fe-S cluster of PS1, O_2_ can be directly reduced by F_B_^−^ although with slow reaction kinetics^45^. In parallel, the current at the Pt microdisk for O_2_ collection (Fig. 3a, blue trace) shows an initial cathodic background current in the dark, corresponding with the steady-state reduction of O_2_ initially present in solution at the Pt microdisk electrode. Upon irradiation, a decrease in the recorded cathodic current indicates the consumption of O_2_ confirming the reduction of O_2_ by PS1. Furthermore, at the Pt microdisk electrode used for the collection of H_2_O_2_ (Fig. 3a, red trace), an increased anodic current was recorded upon irradiation of the sample, thus unequivocally confirming the generation of H_2_O_2_. It is important to note that at the applied potential for O_2_ collection (WE 1, *E*_app_ = −600 mV vs. Ag/AgCl/3 M KCl) partial reduction of H_2_O_2_ at the Pt surface is also possible. Therefore, the obtained net current at this electrode is a sum of the reduction of both O_2_ and H_2_O_2_.

The photocathode was then evaluated in the presence of MV^2+^ (Fig. 3b). As a result of the more efficient uptake of the high energy electrons from PS1 by MV^2+^, an increased photocurrent was now observed (Fig. 3b, gray trace). As expected, the associated current for the collection of O_2_ presented a similar initial background value under dark conditions but a more remarkable decrease was observed upon irradiation (Fig. 3b, blue trace). Moreover, the faster reaction kinetics in the presence of MV^2+^ as primary electron acceptor led to an enhanced production of partially reduced oxygen species, concomitantly with larger anodic currents for the collection of H_2_O_2_ at the corresponding Pt microelectrode (Fig. 3b, red trace). The obtained results clearly provide experimental evidence about the generation of partially reduced ROS at the PS1 photocathode by the in situ collection of H_2_O_2_. The collection of H_2_O_2_ was directly associated with the irradiation of the sample, indicating that reduced oxygen species are exclusively generated under light conditions (Fig. 3a, b). Moreover, the release of H_2_O_2_ was related to the consumption of O_2_ in the gap between the dual microelectrode tip and the sample, confirming that O_2_ in solution is the source of partially reduced oxygen species.

### CuTPA as catalyst for the direct reduction of O_2_ to H_2_O

To verify our observations, we performed a control experiment replacing MV^2+^ by a Cu complex that is able to act as catalyst for the 4 e^−^ reduction of O_2_ to H_2_O, thus preventing any formation of H_2_O_2_. The selected complex, Cu-tris(2-pyridylmethyl)amine nitrate (CuTPA), has been described before as an effective catalyst for the direct reduction of O_2_ to H_2_O (see Supplementary Fig. 2)^46–48^ and exhibited a suitable potential for the uptake of electrons from the F_B_^−^ site of PS1, as determined by cyclic voltammetry (Supplementary Fig. 3). The recorded photocurrent at the sample was of similar magnitude as the one obtained in the presence of MV^2+^, indicating an efficient electron transfer from photo-excited PS1 to the molecular Cu-complex in solution (Fig. 3c, gray trace). The concomitant decrease in the cathodic O_2_ reduction current under irradiation indicated the consumption of O_2_ (Fig. 3c, blue trace). Moreover, and as expected, the production of H_2_O_2_ was prevented when using CuTPA as an electron acceptor (Fig. 3c, red trace).

The small increase in the H_2_O_2_ oxidation current immediately after switching to irradiation may be attributed to the competing reduction of O_2_ by chlorophyll *a* (Chl*a*) (vide infra), leading to the potential formation of H_2_O_2_. This pathway was rapidly overtaken by the reduction of O_2_ and H_2_O_2_ by CuTPA, and hence no significant collection of H_2_O_2_ was further observed. The absence in H_2_O_2_ collection confirmed the previously obtained results and the capability of the analytical system for the determination of H_2_O_2_ as indicator for the presence of partially reduced oxygen species generated under irradiation.

Interestingly, the use of CuTPA as an electron acceptor was observed to lead to a remarkably quicker loss in activity of the PS1/Os-P photocathode as compared with MV^2+^ (Supplementary Fig. 4). The faster decay in photocurrent response in the case of CuTPA could be attributed to Cu-plating at the stromal side of the photosystem. The unavoidable presence of free Cu^2+^ ions in the CuTPA solution entails the direct reduction and deposition of Cu^0^ at the F_B_^−^ site, with the consequent blocking and loss in activity of the photosystem. These results prevent the use of Cu complexes for the development of efficient PS1-based photocathodes working under optimal conditions and with a minimized production of partially reduced oxygen species.

### Evaluation of PS1/Os-P at varying O_2_ concentrations

**Fig. 4:**
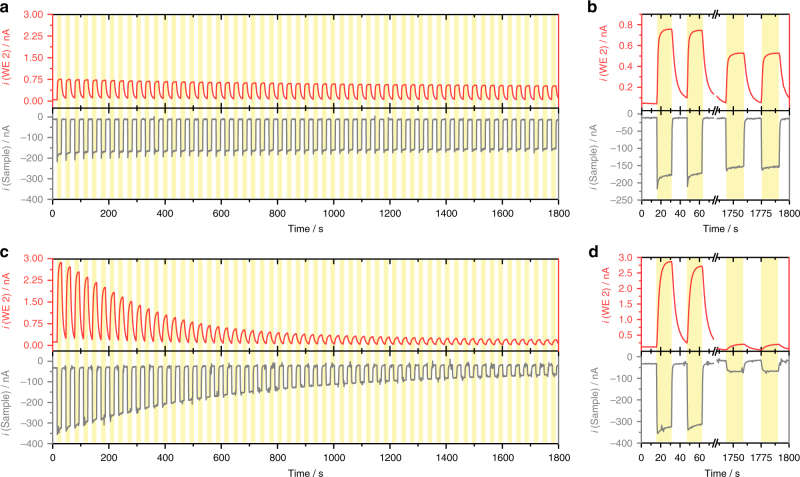
Long-term stability of the photocurrent at varying O_2_ concentrations. Simultaneous photochronoamperometric responses and collection of H_2_O_2_ recorded at a PS1/Os-complex-modified redox polymer photocathode **a**, **b** air-equilibrated solution and **c**, **d** O_2_-saturated solution. The sample was polarized at 0 mV, while the Pt disk microelectrode was polarized at +600 mV vs. Ag/AgCl/3 M KCl (WE 2). Electrolyte: 3 mM MV^2+^ in 0.2 M citrate-phosphate buffer, pH 7.0. Tip-to-sample distance: 10 μm. The sample was locally irradiated (white light, 280 mW cm^−2^) through the sheath of the positioned microelectrode for periods of 15 s as indicated by the yellow shaded regions in the graphs. A comparison of the initially recorded currents with the response obtained at the end of the experiment is given in graphs **b**, **d** (note the different current scales for **b** in comparison with **a**)

The photocurrent and H_2_O_2_ production at the PS1/Os-P photocathode were further evaluated using MV^2+^ as the primary electron acceptor at two different O_2_ concentrations. The PS1/redox polymer photocathode was evaluated in a sequence of successive light and dark periods (see Supplementary Fig. 5). The performance of the photocathode in air-equilibrated solutions was compared with the same experiment in an O_2_-saturated solution. The results are summarized in Fig. 4, where, for the sake of simplicity, the response of the O_2_ reduction at the Pt microdisk polarized at −600 mV (vs. Ag/AgCl/3 M KCl) has been omitted. A relatively stable photocurrent was obtained for the photocathode working in air-equilibrated solution after 30 min (Fig. 4a). In contrast, an increased O_2_ concentration led to a faster loss of activity of the photocathode (Fig. 4c). Comparison of the initial photocurrents with those obtained at the end of the experiment highlights the major loss in activity for photocathodes working under O_2_-saturated solutions (Fig. 4b, d). While in air-equilibrated solution the photocathode retained 87% of the initial response at the end of the experiment, the photocathode working in O_2_-saturated solution only preserved 17% of the initial photocurrent. The faster inactivation in the latter case was associated with a remarkably higher collection of H_2_O_2_ at the Pt microdisk electrode (cf. red traces in Fig. 4a, c).

The long-term stability of the PS1/Os-P film on Au electrodes was confirmed by recording successive cyclic voltammograms under dark conditions and comparing the redox signals of the immobilized polymer over time. The obtained results (Supplementary Fig. 6) exhibited a high stability of the polymer film over at least 100 cycles, ensuring a highly stable film for the duration of the experiments. Moreover, the stability of the redox polymer was also evaluated under conditions that would lead to the generation of ROS in the evaluated system. For this, cyclic voltammograms recorded under dark conditions were compared before and after a long-term uninterrupted photochronoamperometry experiment (Supplementary Fig. 7). The obtained results showed no decrease in the Os^2+^/Os^3+^ redox signal after >40 min of the irradiation period. The slight increase in current observed in the voltammogram recorded after the long-term experiment could be ascribed to the swelling of the hydrogel due to solvation of the film. Although the film was exposed to the reactive species causing a loss in activity observed in the recorded photocurrent, the Os-complex-modified redox polymer was sufficiently stable, confirming that the loss in activity of the photocathode was not attributed to a damage of the film or loss of material from the electrode surface but had to be related instead to an inactivation of the immobilized photosynthetic protein complexes.

Furthermore, to confirm that O_2_ was involved in the loss of activity over time of the evaluated photoelectrode through the generation of ROS under irradiation, a long-term photochronoamperometric study of the PS1/Os-P assembly was performed under nearly anaerobic conditions. In this case, 2-methyl-1,4-naphthoquinone was used as an electron acceptor for the uptake of electrons from the F_B_ site of PS1 in absence of O_2_ in solution. The obtained results (Supplementary Fig. 8) exhibited an increased stability of the analyzed photocathode in comparison with the system evaluated under aerobic conditions and using MV^2+^/O_2_ as an electron acceptor (cf. Supplementary Fig. 9 and Fig. 1). Under anaerobic conditions, the photocathode retained about 96% of the initial response after 30 min of the experiment, thus surpassing the previously observed retained activity after the same period of time for the photocathode evaluated under air-equilibrated solutions. This fact clearly confirmed the implication of O_2_-derived reactive species that are responsible for the limited stability of the biophotocathode.

### Incorporation of Cat and SOD

Following nature’s scavenging mechanisms developed to overcome the deleterious effects of commonly generated ROS in living organisms, the enzymes SOD and Cat were selected for the suppression of harmful effects caused by oxygen radicals to the PS1/Os-complex-modified redox polymer photocathode. The enzymes were first co-immobilized together with PS1 within the redox polymer film. Different electrodes were fabricated including Cat, SOD, or a mixture of both enzymes together with the PS1/Os-P film deposited on Au electrode surfaces. The recorded current for the collection of H_2_O_2_ exhibited a significant decrease only in the presence of relatively large amounts of Cat in the polymer film (Supplementary Fig. 10). The fact that the photocurrent magnitude was similar for electrodes containing co-immobilized Cat or SOD confirmed that the same amount of PS1 was present in the mixed hydrogel film and that the photocathode response was not affected by the presence of Cat or SOD in the film. Further removal of produced H_2_O_2_ was not possible without affecting the magnitude of the obtained photocurrents. The effective removal of H_2_O_2_ by the disproportionation reaction catalyzed by Cat was evaluated by depositing a thin film of Cat or denatured Cat over the PS1/Os-P assembly. The obtained results (see Supplementary Fig. 11) showed that most of the generated H_2_O_2_ was consumed by the active immobilized enzyme before reaching the microelectrode. In contrast, when denatured Cat was used, H_2_O_2_ was able to diffuse through the film without being consumed, reaching the microelectrode where it was finally detected. Since the incorporation of a large amount of enzymes within the redox polymer disrupts the properties of the film in terms of electron transfer and diffusion rates (see Supplementary Fig. 12), unambiguous conclusions cannot be obtained about the long-term stability of the evaluated photocathode.

Further experiments with the enzymes Cat and SOD in solution indicated a clearly diminished collection of H_2_O_2_ as compared with the PS1-based photocathode in the absence of Cat or SOD (Supplementary Fig. 13). However, long-term measurements of the photocurrent for photocathodes working with Cat and SOD in solution revealed that even in the presence of scavenging enzymes the stability of the photocathode could not be increased. At the end of the experiment, the remaining photocurrent was about 62% with SOD, 82% with Cat, and 85% with the mixture of enzymes; compared to 87% for the PS1 photocathode in the absence of these enzymes (Supplementary Fig. 13). The obtained results suggested that the production of reactive species takes place within the protein complex or in close proximity to it. Hence, the generated species react rapidly before their harmful effect can be prevented by any scavenging mechanism that require the diffusion of large scavenger species.

### Partially reduced oxygen species produced by Chl*a*

**Fig. 5:**
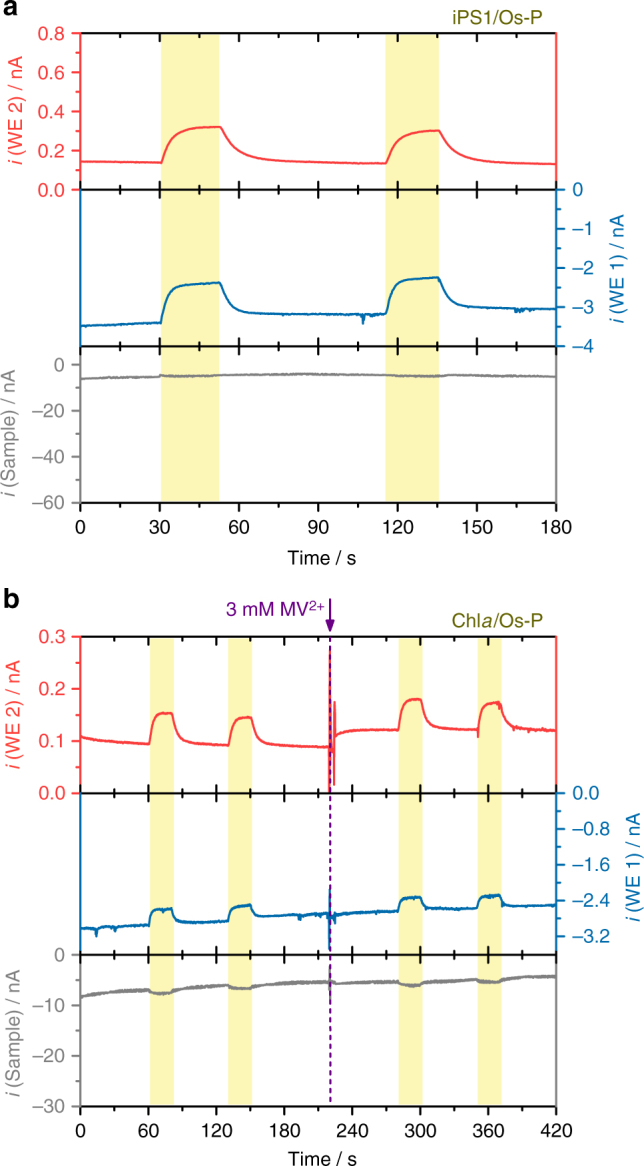
Production of partially reduced oxygen species at inactivated PS1 and free Chl*a* under irradiation. Simultaneous photochronoamperometric responses recorded for **a** iPS1/Os-P and **b** free Chl*a*/Os-P-modified electrodes. Electrolyte: 3 mM MV^2+^ in 0.2 M citrate-phosphate buffer, pH 7.0 (for **b** addition of MV^2+^ at 220 s). The sample was polarized at 0 mV, while the Pt disk microelectrodes were polarized at −600 mV (WE 1) and +600 mV (WE 2). All potentials vs. Ag/AgCl/3 M KCl. Tip-to-sample distance: 10 μm. The sample was locally irradiated (white light, 280 mW cm^−2^) for periods of 20 s, as indicated by the yellow shaded regions in the graphs

In order to evaluate whether the generation of H_2_O_2_ as collected at the SPECM microelectrode tip was only associated with the main pathway of light-induced charge separation at PS1 (Fig. 1), a PS1/Os-complex-modified redox polymer photocathode was analyzed using PS1 that had been previously inactivated by thermal treatment (see Methods section). The obtained results in the absence of any redox mediator in solution (Fig. 5a) revealed the generation of H_2_O_2_ even using the inactivated PS1 under irradiation (Fig. 5a, red trace) while the absence of any significant photocurrent confirmed the complete absence of iPS1 activity (Fig. 5a, gray trace). As described previously for PS2, chlorophyll molecules may be involved in the direct photo-induced reduction of O_2_^42^. After thermal treatment, the PS1 protein complex was not anymore in a fully functional state, and in consequence, the capability to perform light-induced charge separation was suppressed. Under these conditions, the tridimensional structure of the complex was lost, leaving exposed chlorophyll molecules accessible for electron-transfer processes and enabling the direct reduction of O_2_ at the chlorophyll upon light absorption. This led to the generation of partially reduced oxygen species and was accompanied by the formation of H_2_O_2_ as detected at the SPECM tip. Further studies were performed with respect to O_2_ reduction by chlorophyll pigments under irradiation. For this, a film of free Chl*a* embedded in the same Os-complex-modified redox polymer and deposited on an Au electrode surface was analyzed. The results clearly showed the formation of H_2_O_2_ collected at the SPECM tip under irradiation of the sample (Fig. 5b), confirming that Chl*a* was capable for the reduction of O_2_ upon irradiation. Addition of MV^2+^ did not lead to significant differences, indicating that MV^2+^ was not involved in this reaction and that the excited chlorophyll directly reduced O_2_ in solution (notice the decreased cathodic current indicating O_2_ consumption under irradiation, Fig. 5b, blue trace).

## Discussion

We have demonstrated the in situ detection of H_2_O_2_ and the associated O_2_ consumption at PS1/redox polymer-based photocathodes under light stress conditions. Performing the experiments over long periods of time while working with intermittent consecutive dark and light conditions were used to minimize variations in electron transfer and diffusion rates by potential local heating effects. Moreover, the use of intermittent illumination allows recording a clear baseline for photocurrent monitoring over extended periods of time, making long-term evaluation of the bioelectrodes under different conditions possible. PS1/Os-complex-modified redox polymer photocathodes working under aerobic conditions generate partially reduced oxygen species, which are responsible for the degradation of the PS1 protein complex and a drop in activity of the photoelectrodes with time. Incorporation of Cat and SOD in the PS1/Os-P biodevice demonstrates that suppression of partially reduced oxygen species is difficult due to the high reactivity and short diffusion time of partially reduced oxygen species. We have additionally shown that the formation of these partially reduced reactive species under light is not only associated with the main electron-transfer pathway following charge separation by PS1 upon light absorption. H_2_O_2_ associated with O_2_ consumption could be also demonstrated for thermally inactivated PS1 complexes and free Chl*a*, indicating the capability of O_2_ reduction by chlorophyll pigments. Our findings provide further understanding about electron-transfer pathways at PS1-modified electrode surfaces and highlight the limited applicability of PS1-based photocathodes working under aerobic conditions, where the unavoidable formation of ROS is responsible for an overall short long-term stability. Hence, the obtained results are strongly encouraging the design of PS1-based biophotocathodes operating under strictly anaerobic conditions.

## Methods

### Chemicals and materials

Poly(ethylene glycol)diglycidyl ether (PEGDGE, Polysciences), methyl viologen dichloride hydrate (Sigma-Aldrich, 98%), and 2-methyl-1,4-naphthoquinone (Alfa Aesar, 98%) were of reagent grade and used without further purification. The redox polymer poly(1-vinylimidazole-*co*-allylamine)-Os(2,2′-bipyridyl)_2_Cl was synthesized and purified following previously reported procedures^13, 49^. Briefly, a mixture of 4.6 mmol allylamine and 30.6 mmol vinylimidazole was copolymerized under argon atmosphere. The reaction was initiated by 1.5 mmol of 2,2′-azobisisobutyronitrile and stirred for 2 h at 70 °C. The product was dissolved in ethanol, filtered, washed with acetone, and dried in vacuum. The copolymer backbone (84 mg mL^−1^ in ethanol) was then allowed to react with 0.18 mmol [Os(bipy)_2_Cl_2_] at 90 °C under continuous stirring to give the final product. Cat from bovine liver (EC 1.11.1.6) and SOD from bovine erythrocytes (EC 1.15.1.1) were from Sigma-Aldrich. PS1 was isolated from cultures of the thermophilic cyanobacterium *Thermosynechococcus elongatus* BP-1 as described elsewhere^45^. In brief, the cells were grown in a 20 L foil fermenter at 45 °C in BG11 medium supplemented with 5% CO_2_ in air and light of increasing intensity (20 to 100 µmol photons m^−2^ s^−1^). Cells were harvested at an OD_680_ of 3–3.5 and disrupted by Parr bomb. PS1 was isolated from thylakoid membranes by β-dodecyl maltoside treatment (1.2%) followed by hydrophobic interaction chromatography and ion exchange chromatography. Purified PS1 showed an oxygen consumption activity of about 1100 µmol O_2_ h^−1^ mg_Chl_^−1^. The inactivated photosystem (iPS1) was prepared by thermal treatment of PS1^50^ using a silicone oil bath at 120 °C for 60 min. Chl*a* was obtained from the purified PS1 protein sample by incubation for 24 h at room temperature (RT) in absolute EtOH (ThermoFisher Scientific), followed by centrifugation (15,500 × *g*, RT, 10 min). The supernatant was concentrated by centrifugal vacuum (Savant DNA 110 SpeedVac, ThermoFisher Scientific). All solutions were prepared with deionized water (*ρ* = 18 MΩ cm).

### Electrode modification

Gold disk electrodes (2 mm Ø, BASi) were cleaned prior to modification by polishing with diamond suspension (LECO) of decreasing grain sizes (0.3 µm, 0.1 µm, 0.05 µm) and then sonicated successively in absolute ethanol and water. The gold electrodes were then electrochemically cleaned by cyclic voltammetry, recording 20 scans from −0.1 to 1.6 V (vs. Ag/AgCl/3 M KCl) in 0.5 M H_2_SO_4_ at 100 mV s^−1^. The electrode surface was modified with 2.5 µL of a mixture of the redox polymer (5 µg µL^−1^), PEGDGE (0.02 µg µL^−1^), and the isolated PS1 (1 µg µL^−1^) or free Chl*a* (amount normalized by the chlorophyll content of PS1). PS1, iPS1, and Chl*a* samples were incubated overnight in the dark at 4 °C. Before measurement, the modified electrodes were incubated for 30 min in 50 mM Tris-HCl buffer solution of pH 9 (containing 100 mM KCl, 10 mM MgCl_2_, and 10 mM CaCl_2_) to induce polymer collapse and crosslinking^18^.

For the evaluation of films including Cat and SOD, Au-coated Si wafers were used as sample electrodes. Si(100) wafers (Wacker) were coated by vapor deposition in a metal vaporization setup (Leybold Univex 300) with a 1000 Å layer of gold over a 50 Å adhesion layer of titanium. The Au wafers were cleaned with Piranha solution and then rinsed with distilled water before modification. The substrates were modified with 4 µL of a mixture of the redox polymer (5 µg µL^−1^), PEGDGE (0.02 µg µL^−1^), and the isolated PS1 (1 µg µL^−1^). Immediately after, an aliquot of Cat (5 µg µL^−1^) and/or SOD (5 µg µL^−1^) solution was dropped on the modified electrode, gently mixed, and incubated overnight to achieve the combined film.

### Synthesis of CuTPA

CuTPA (C_18_H_18_CuN_6_O_6_, Supplementary Fig. 2, molecular weight: 477.92 g mol^−1^) was synthesized as follows. Tris(2-pyridylmethyl)amine (Sigma-Aldrich, 62.4 mg; 0.2 mmol) was dissolved in absolute EtOH (11 mL). Cu(NO_3_)_2_·3H_2_O (25.4 mg; 0.1 mmol, 0.5 eq.) was added and the mixture was stirred at reflux temperature for 30 min. EtOH was later removed by rotary evaporation. The crude product was washed with diethyl ether (3× 3 mL) and dried under vacuum offering the final product CuTPA in quantitative yield.

### Characterization of electrochemical probes

Dual platinum microdisk electrodes were used for the simultaneous collection of O_2_ and H_2_O_2_ as well as for the localized irradiation of the investigated sample. The electrodes consisted of two platinum wires (25 μm Ø, Goodfellow) sealed in a theta-type borosilicate glass capillary (Supplementary Fig. 1a). Cyclic voltammetry was used to investigate the electrochemical behavior of the dual electrode tips and to determine the surface area of the Pt microdisk electrodes (Supplementary Fig. 1b). The surface area was (5.2 ± 0.8) × 10^−6^ cm^2^. Electrochemical crosstalk between the micro-electrodes originating from the overlap of the steady-state diffusion profiles formed at the two microdisk electrodes was evaluated by performing generation–collection experiments. For this, one of the disks was swept in a conventional cyclic voltammetric experiment, while the other disk was held at a potential sufficiently high to achieve steady-state oxidation of the reduced species generated at the other disk (Supplementary Fig. 1c,d). The fabricated electrodes exhibited different distances between the two microdisk surfaces (Supplementary Fig. 1a). Electrodes with a minimum separation of 60 µm were selected, corresponding to a crosstalk of <5% and ensuring minimal interference between the measured currents at both microdisk electrodes.

### Photochronoamperometric measurements

SPECM measurements were performed using the modified gold electrode as sample, the dual Pt microdisk electrode as tip, an Ag/AgCl/3 M KCl reference electrode, and a Pt cylindrical mesh as counter electrode. The SPECM setup was previously described^42^ and consisted of step-motor-driven micrometer screws (Owis) and piezoelectric positioners (NanoCube, Physik Instrumente) for accurate positioning of the tip electrode in *x*–*y*–*z* directions, a bipotentiostat (PGU-BI 100, IPS-Jaissle), and an in-house written control software. The main channel of the bipotentiostat was connected to the sample electrode while the second channel was connected to one of the Pt microdisk electrodes (Tip WE 1). A coupled Petit Ampère potentiostat (BASi) was used for the third channel and connected to the other Pt microdisk electrode (Tip WE 2). For local illumination of the sample, a Hg–Xe lamp (LC8 type 03, Hamamatsu Photonics) was coupled with the top glass wall of the tip microelectrode by means of a light fiber (HITRONIC POF Simplex PE). The dual Pt microdisk electrode was used simultaneously as a probe for electrochemical interrogation of the sample as well as for a source for localized illumination. The samples were irradiated using white light (Supplementary Fig. 14) with an effective light intensity of 280 mW cm^−2^ reaching the sample surface.

Photochronoamperometric measurements were performed with the tip positioned at a constant distance of 10 μm above the surface of the interrogated sample in a stationary configuration. The tip was precisely positioned above the sample by recording approach curves in buffer solution keeping the sample unbiased and the tip microelectrode polarized at −600 mV, a potential sufficient for the steady-state reduction of O_2_ in solution. Simultaneous collection of O_2_ and H_2_O_2_ was performed by polarizing one of the Pt microdisk electrodes at −600 mV, whereas the other was held at +600 mV. The sample potential was kept constant at an applied potential of 0 mV. All indicated potentials are vs. Ag/AgCl/3 M KCl.

All experiments were performed at a temperature of (22 ± 1) °C.

### Data availability

Data supporting the findings of this manuscript are available from the corresponding authors upon reasonable request.

## Electronic supplementary material

Supplementary Information
