# Supplementary material for: Light-induced formation of partially reduced oxygen species limits the lifetime of photosystem 1-based biocathodes
Source: Nat Commun. 2018 May 17;9:1973. doi: 10.1038/s41467-018-04433-z (PMC5958124; doi:10.1038/s41467-018-04433-z)
Supplement: Supplementary file 1 — Supplementary Information [file 41467_2018_4433_MOESM1_ESM.pdf]

Supplementary Information for the Manuscript:

“Light-induced formation of partially reduced oxygen species limits the lifetime of photosystem 1-based biocathodes”

Zhao et al.

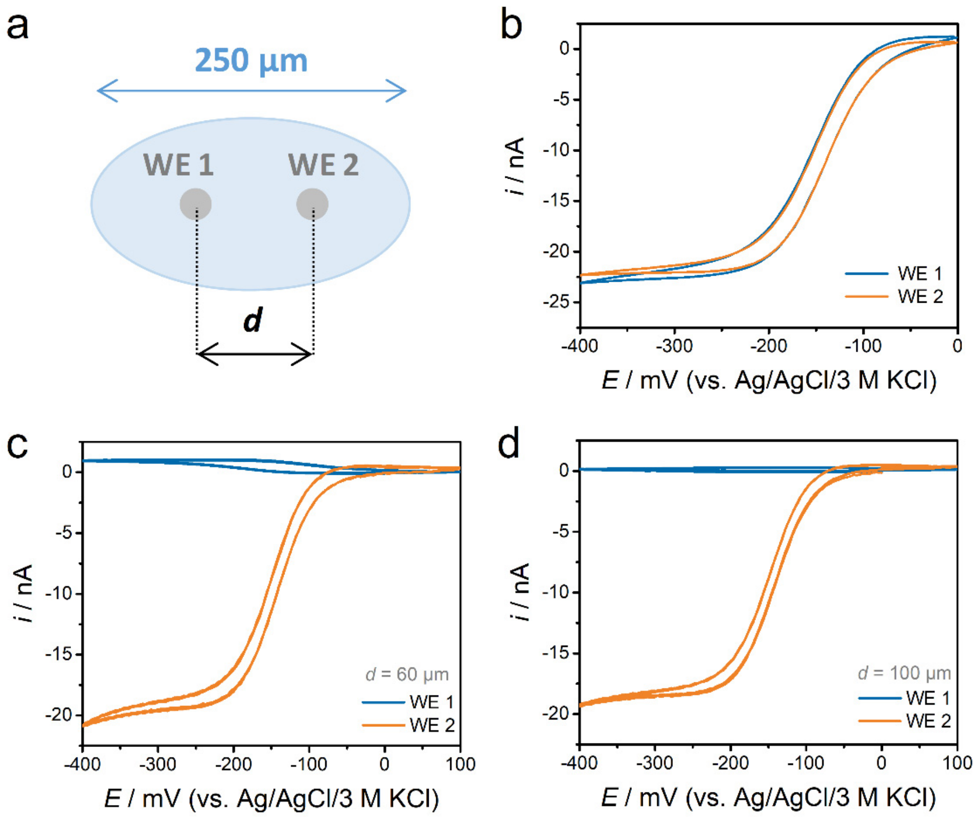

Supplementary Fig. 1. Characterization of the dual microelectrode SPECM tips. (**a**) Schematic representation of the geometry of the dual Pt tip. The microdisk electrodes are separated by a distance *d*. (**b**) Cyclic voltammograms recorded with the dual Pt microdisk electrodes for the determination of the surface area. Electrolyte: 5 mM $[Ru(NH_3)_6]^{3+}$ in 0.1 M KCl. Scan rate = 50 mV s^{-1}. (**c**,**d**) Generation-collection experiment for the evaluation of cross-talk influence at tips fabricated with the microdisk electrodes separated by a different distance *d* according to the scheme in panel (a). WE 2 (generator electrode): scan rate = 20 mV s^{-1}; WE 1 (collector electrode): E_{app} = 100 mV (vs. Ag/AgCl/3 M KCl). Electrolyte: 5 mM $[Ru(NH_3)_6]^{3+}$ in 0.1 M KCl.

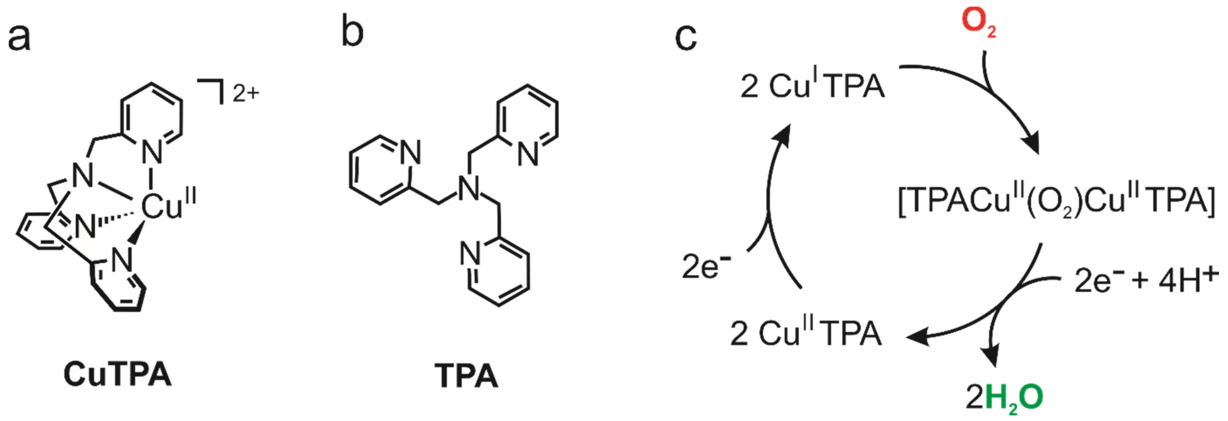

Supplementary Fig. 2. CuTPA as catalyst for the net 4 e$^-$ reduction of O_2 to water. (**a**) Structure of the Cu-tris(2-pyridylmethyl)amine complex (CuTPA). (**b**) Structure of the tetradentate ligand tris(2-pyridylmethyl)amine (TPA). (**c**) Catalytic cycle for O_2 reduction. Two CuTPA complexes coordinate side-to-side to the O_2 molecule. The complex is prone to proton promoted reductive O-O cleavage, leading to water and the regeneration of the catalyst[1,2].

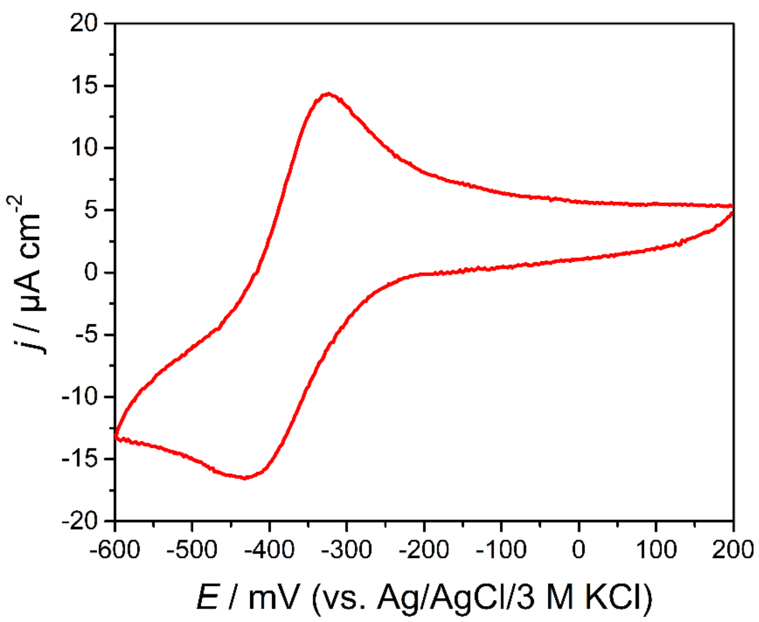

Supplementary Fig. 3. Electrochemical characterization of CuTPA by cyclic voltammetry for the determination of the redox potential of the complex. Glassy carbon working electrode, Ag/AgCl/3 M KCl reference electrode and Pt-counter electrode. Electrolyte: 100 µM CuTPA in Ar-saturated 0.1 M phosphate buffer, pH 7.5. Scan rate = 100 mV s^{-1}.

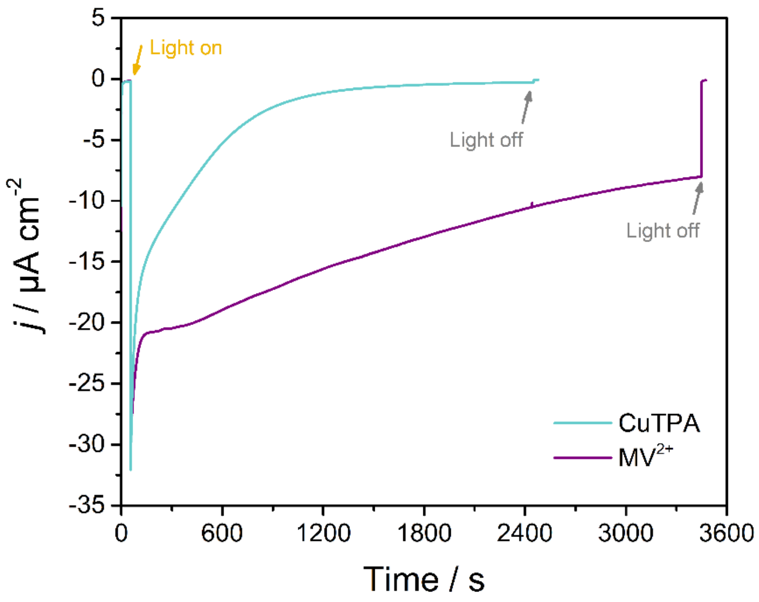

Supplementary Fig. 4. Comparison of long-term photochronoamperometric responses for PS1/Os-polymer biocathodes using MV^{2+} or CuTPA as electron acceptor in air-equilibrated solutions. Electrolyte: 3 mM MV^{2+} or 3 mM CuTPA in 0.2 M citrate-phosphate buffer, pH 7.0. E_{sample} = 0 mV (vs. Ag/AgCl/3 M KCl). The electrodes were globally irradiated using a 685 nm LED with an incident power of 40 mW cm^{-2} for different periods, as indicated in the graph. Note the much faster decay in photocurrent for CuTPA in comparison with MV^{2+}.

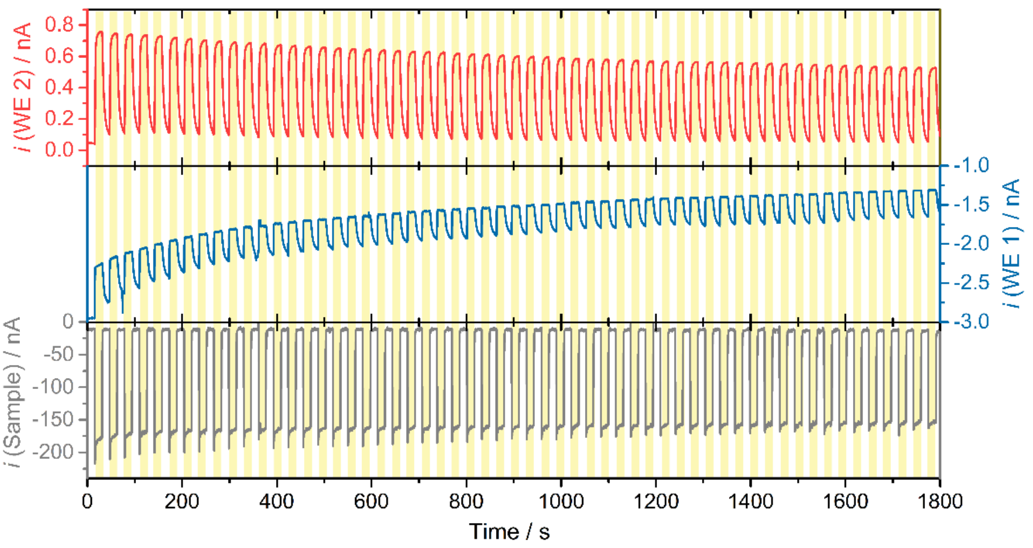

Supplementary Fig. 5. Simultaneous photochronoamperometric responses recorded during the long-term evaluation of a PS1/Os-P photocathode on a gold electrode substrate (sample) using a dual Pt microelectrode tip. The sample was polarized at 0 mV, while the Pt disk microelectrodes were polarized at −600 mV (WE 1) and +600 mV (WE 2), in all cases vs. Ag/AgCl/3 M KCl. Electrolyte: 3 mM MV^{2+} in 0.2 M citrate-phosphate buffer, pH 7.0. Tip-to-sample distance: 10 µm. The analyzed sample was locally irradiated (white light, 280 mW cm^{-2}) for periods of 15 s, as indicated by the yellow shaded regions in the graph.

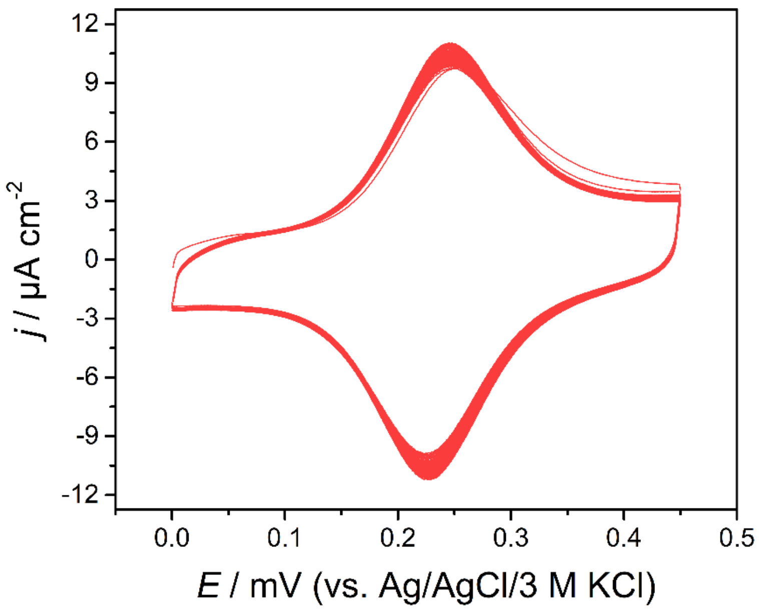

Supplementary Fig. 6. Long-term stability of the PS1/Os-complex modified redox polymer film deposited on an Au electrode substrate. 100 consecutive cyclic voltammograms recorded under dark. Electrolyte: 3 mM MV^{2+} in 0.2 M citrate-phosphate buffer, pH 7.0. Scan rate = 50 mV s^{-1}.

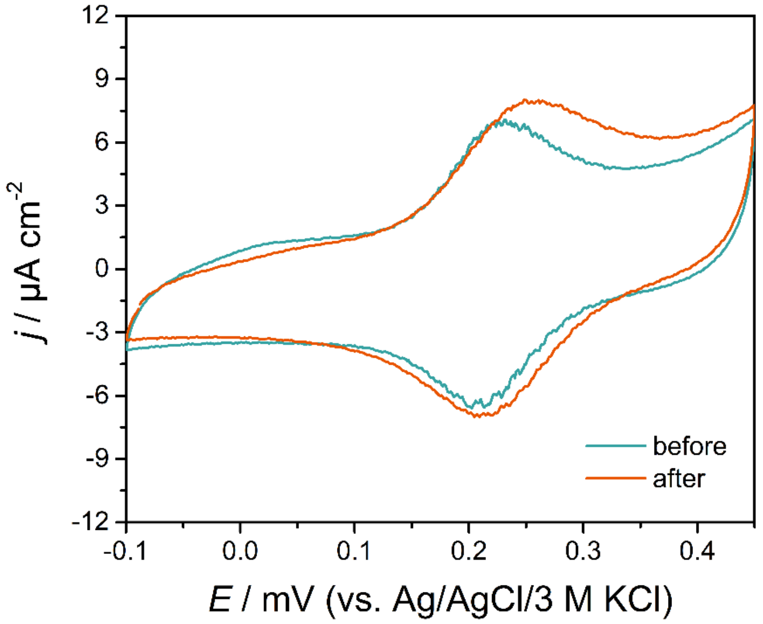

Supplementary Fig. 7. Evaluation of the stability of the Os-complex modified redox polymer film exposed to reactive oxygen species generated under irradiation. Cyclic voltammograms recorded under dark for a PS1/Os-P modified Au electrode before and after an irradiation period of over 40 min. Experimental conditions as in Supplementary Fig. 5. Electrolyte: 3 mM MV^{2+} in 0.2 M citrate-phosphate buffer, pH 7.0. Scan rate = 50 mV s^{-1}.

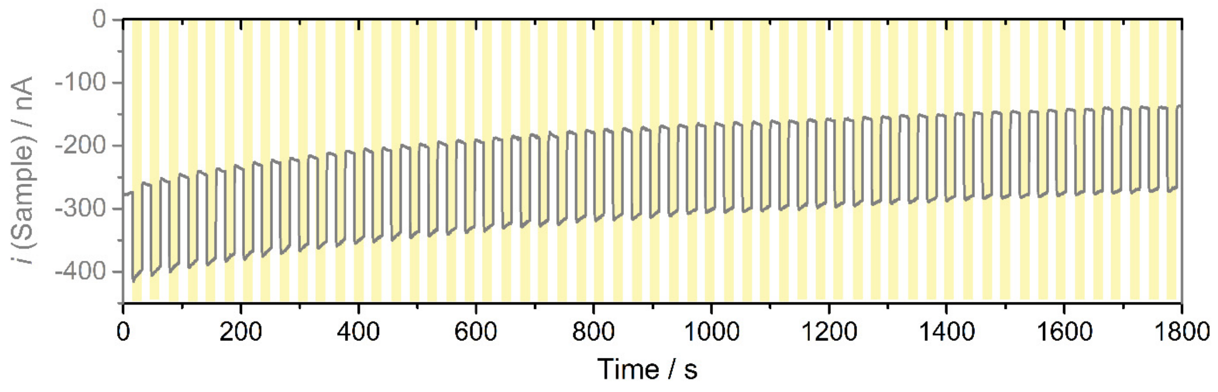

Supplementary Fig. 8. Photochronoamperometric response recorded during the long-term evaluation of a PS1/Os-P photocathode on a gold electrode substrate (sample) under anaerobic conditions. The sample was polarized at 20 mV vs. Ag/AgCl/3 M KCl. Electrolyte: Ar-saturated 0.5 mM methyl naphthoquinone in 0.2 M sodium citrate buffer, pH 4.0. Tip-to-sample distance: 10 µm. The analyzed sample was locally irradiated (white light, 280 mW cm^{-2}) for periods of 15 s, as indicated by the yellow shaded regions in the graph. The absence of O_2 implies the presence of reduced charge carriers in solution preventing to monitor the possible generation of H_2O_2.

a

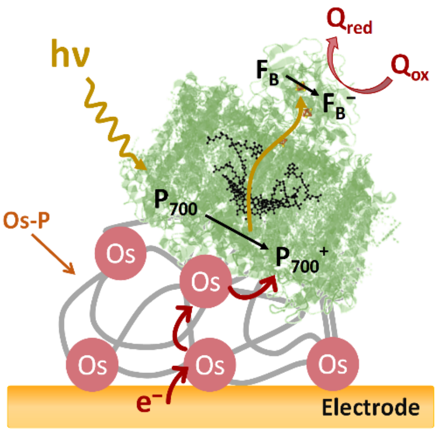

b

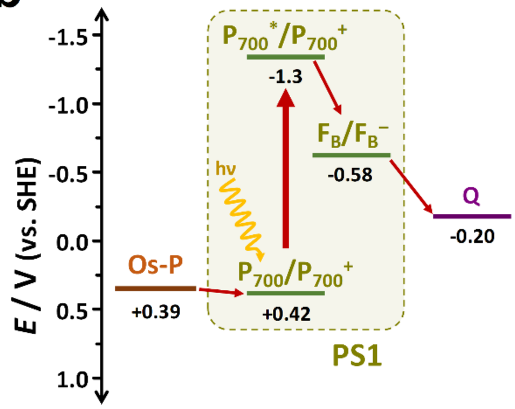

Supplementary Fig. 9. (**a**) Schematic representation of the PS1 photocathode evaluated under anaerobic conditions. 2-Methyl-1,4-naphthoquinone is used as electron acceptor in solution for the uptake of electrons from the reduced Fe-S cluster F_B^- at the stromal side of PS1. (**b**) Energy levels for the terminal cofactors involved in the electron transfer sequence of PS1. Formal potential of 2-methyl-1,4-naphthoquinone (Q) at pH 4.0 (25 °C).

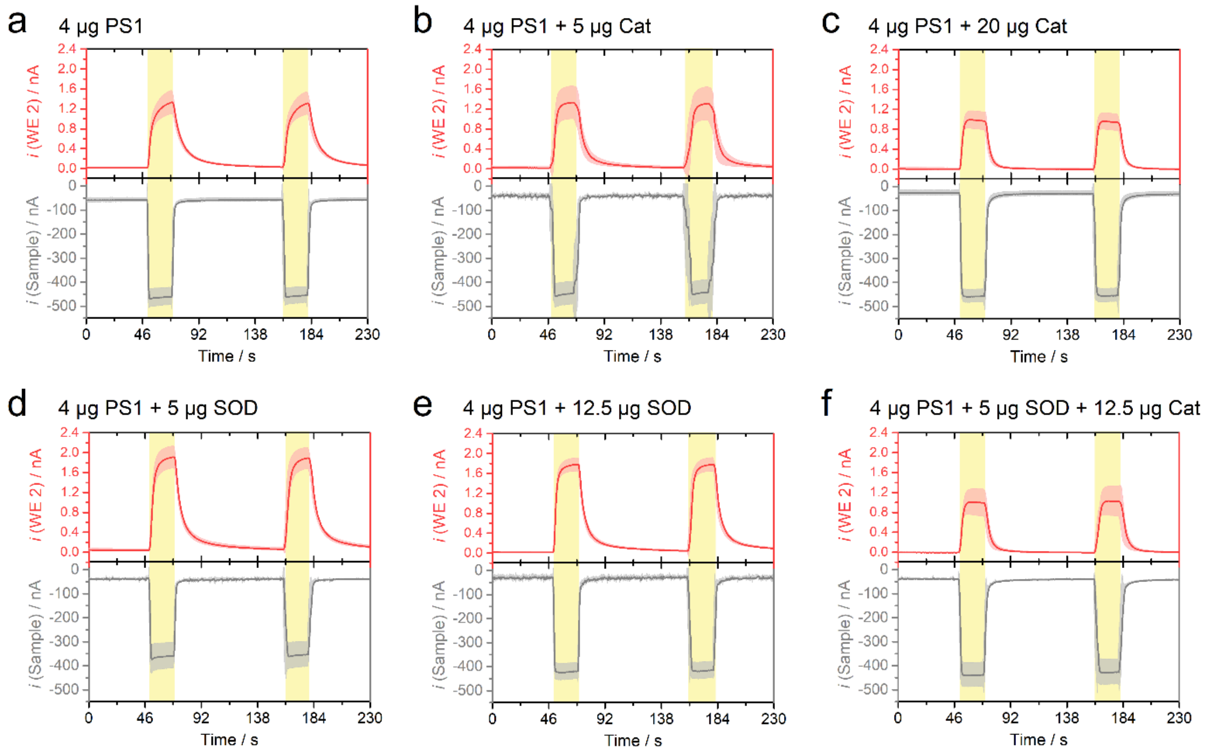

Supplementary Fig. 10. Immobilization of Cat and SOD within the redox polymer film. Mean photochronoamperometric current recorded at the sample (gray traces) and collection of H_2O_2 (WE 2, red traces) obtained for a PS1/Os-P photocathode (**a**) and with photocathodes prepared including catalase (**b,c**), superoxide dismutase (**d,e**) or a mixture of both enzymes (**f**) in the polymer film. The shaded regions indicate one standard deviation of the measurements ($n = 6$). The composition of the film is indicated for each panel as the amount of biomolecules in the solution deposited on the gold electrode substrate. The sample was polarized at 0 mV, while the Pt disk microelectrode was polarized at +600 mV (WE 2), both vs. Ag/AgCl/3 M KCl. Electrolyte: 3 mM MV^{2+} in 0.2 M citrate-phosphate buffer, pH 7.0. Tip-to-sample distance: 10 µm. The analyzed sample was locally irradiated (white light, 280 mW cm^{-2}) for periods of 20 s, as indicated by the yellow shaded regions in the graphs.

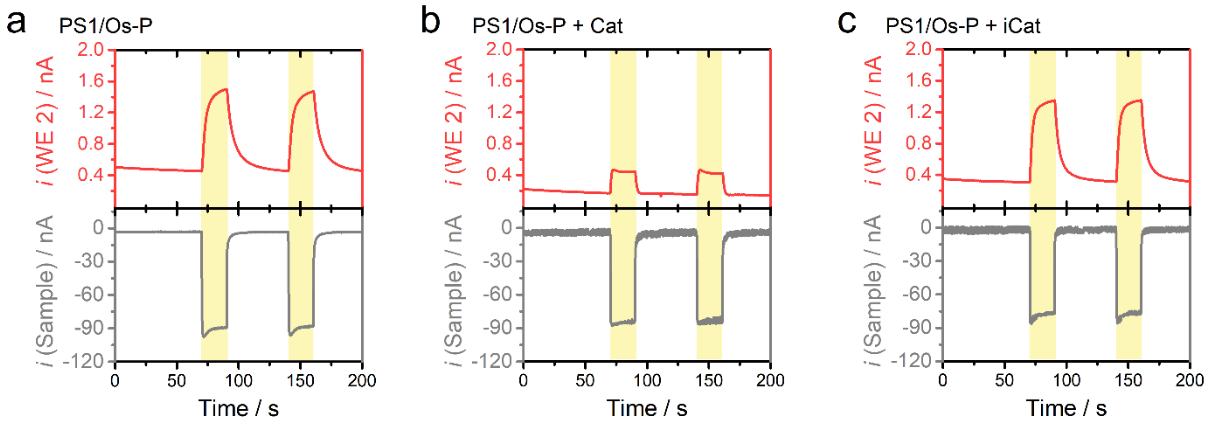

Supplementary Fig. 11. Evaluation of enzymatic activity for the effective removal of H_2O_2. Current recorded at the sample (gray traces) and collection of H_2O_2 (WE2, red traces) obtained for a PS1/Os-P photocathode (**a**) and with the same photocathode bearing a top layer of catalase (**b**) or denatured catalase (**c**). The sample was polarized at 0 mV, while the Pt disk microelectrode was polarized at +600 mV (WE 2), both vs. Ag/AgCl/3 M KCl. Electrolyte: 3 mM MV^{2+} in 0.2 M citrate-phosphate buffer, pH 7.0. Tip-to-sample distance: 10 µm. The analyzed sample was locally irradiated (white light, 280 mW cm^{-2}) for periods of 20 s, as indicated by the yellow shaded regions in the graphs.

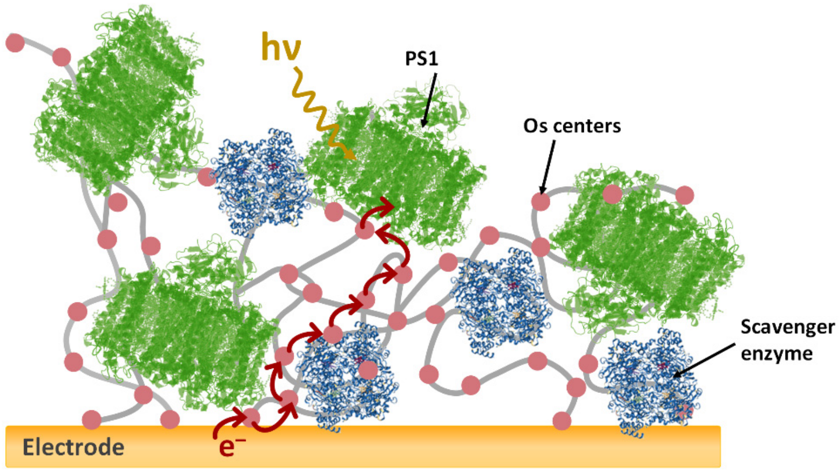

Supplementary Fig. 12. Schematic representation of the PS1 photocathode with scavenger enzymes embedded within the redox polymer film. The presence of a large amount of immobilized enzymes affects electron transfer rates and the diffusion of species within the film.

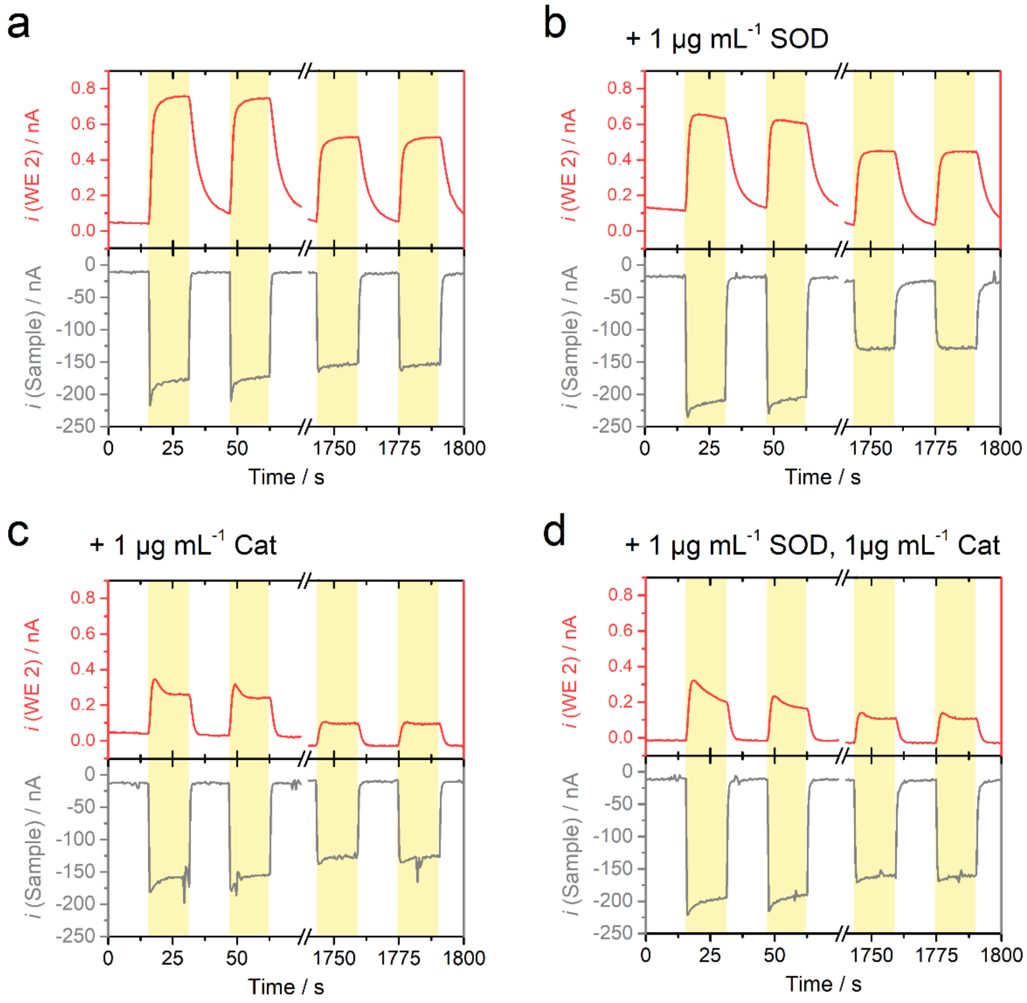

Supplementary Fig. 13. Cat and SOD in solution. Comparison of initial and final responses obtained during a long-term experiment for the current recorded at the sample (gray traces) and collection of H_2O_2 (WE 2, red traces) for a PS1/Os-P photocathode on a gold electrode substrate (**a**). The same experiment was performed with superoxide dismutase (**b**), catalase (**c**) or both enzymes (**d**) in the electrolyte solution (concentration indicated at each corresponding panel). The sample was polarized at 0 mV, while the Pt disk microelectrode was polarized at +600 mV (WE 2), both vs. Ag/AgCl/3 M KCl. Electrolyte: 3 mM MV^{2+} in 0.2 M citrate-phosphate buffer, pH 7.0. Tip-to-sample distance: 10 µm. The analyzed sample was locally irradiated (white light, 280 mW cm^{-2}) for periods of 15 s, as indicated by the yellow shaded regions in the graphs.

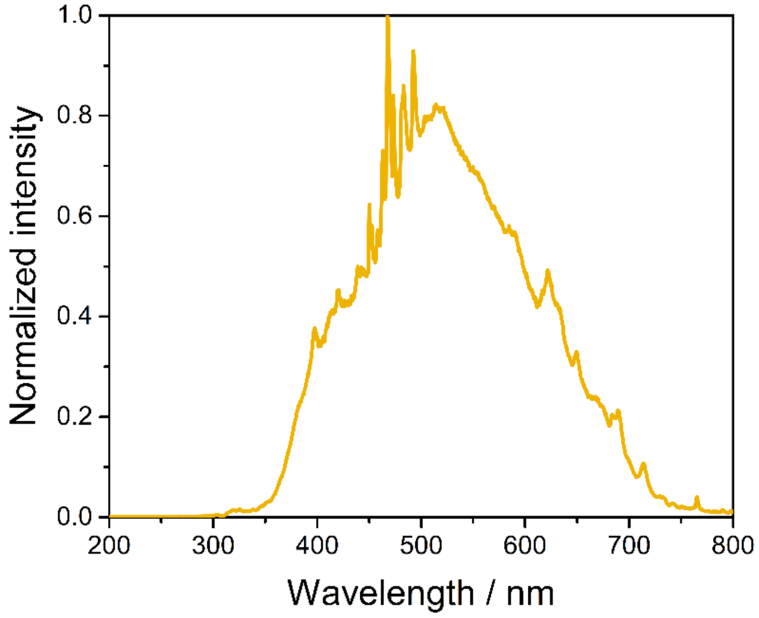

Supplementary Fig. 14. Irradiation spectrum of the light source collected at the output of the glass microelectrode used for local illumination of the sample. The intensity was normalized at each wavelength with respect to the maximum.

Supplementary References

1. Tahsini, L. *et al.* Electron-transfer reduction of dinuclear copper peroxo and bis-mu-oxo complexes leading to the catalytic four-electron reduction of dioxygen to water. *Chem. Eur. J.* **18,** 1084–1093 (2012).
2. Kakuda, S., Peterson, R.L., Ohkubo, K., Karlin, K.D. & Fukuzumi, S. Enhanced catalytic four-electron dioxygen (O_2) and two-electron hydrogen peroxide (H_2O_2) reduction with a copper(II) complex possessing a pendant ligand pivalamido group. *J. Am. Chem. Soc.* **135,** 6513–6522 (2013).
